# Selenoprotein K enhances STING oligomerization to facilitate antiviral response

**DOI:** 10.1371/journal.ppat.1011314

**Published:** 2023-04-06

**Authors:** Lin Lv, Li Chai, Jie Wang, Mengge Wang, Danhui Qin, Hui Song, Yue Fu, Chunyuan Zhao, Jihui Jia, Wei Zhao, Mutian Jia

**Affiliations:** 1 Key Laboratory for Experimental Teratology of the Chinese Ministry of Education, and Key Laboratory of Infection and Immunity of Shandong Province, School of Basic Medical Science, Cheeloo College of Medicine, Shandong University, Jinan, Shandong, China; 2 Department of Physiology & Pathophysiology, School of Basic Medical Science, Cheeloo College of Medicine, Shandong University, Jinan, Shandong, China; 3 Department of Cell Biology, School of Basic Medical Science, Cheeloo College of Medicine, Shandong University, Jinan, Shandong, China; Florida State University, UNITED STATES

## Abstract

Stimulator-of-interferon gene (STING) is a vital element of the innate immune system against DNA viruses. Optimal activation of STING is crucial for maintaining immune homeostasis and eliminating invading viruses, and the oligomerization of STING is an essential prerequisite for STING activation. However, the mechanism of cGAMP-induced STING oligomerization in ER remains unclear. Selenoproteins are crucial for various physiological processes. Here, we identified that the endoplasmic reticulum (ER)-located transmembrane selenoprotein K (SELENOK) was induced during virus infection and facilitated innate immune responses against herpes simplex virus-1 (HSV-1). Mechanistically, SELENOK interacts with STING in the ER and promotes STING oligomerization, which in turn promotes its translocation from the ER to the Golgi. Consequently, *Selenok* deficiency suppresses STING-dependent innate responses and facilitates viral replication *in vivo*. Thus, the control of STING activation by selenium-mediated SELENOK expression will be a priming therapeutic strategy for the treatment of STING-associated diseases.

## Author summary

Selenoproteins are crucial for various physiological processes. Most selenoproteins locate in the cytoplasm and regulate redox pathways to protect cells from oxidative stress. However, the potential roles of transmembrane selenoproteins are largely unknown. Here, we identified Selenoprotein K (SELENOK), an ER-located transmembrane selenoprotein, as a physiological enhancer of STING-dependent antiviral innate immunity. SELENOK is upregulated during viral infection and enhances STING oligomerization to facilitate the activation of the cGAS-STING pathway and suppress viral replication. These results will be valuable for understanding the physiological roles of transmembrane selenoproteins in immunity. Furthermore, as aberrant STING activation has been linked to various pathological conditions, control of STING activity by SELENOK may be a prime therapeutic strategy.

## Introduction

Stimulator-of-interferon gene (STING) is a vital adaptor in the innate immune signaling, which is activated by the second messenger cyclic guanosine monophosphate (GMP)-adenosine monophosphate (AMP) (cGAMP) [1–4]. The key cytosolic DNA sensor cyclic GMP–AMP (cGAS) recognizes cytoplasmic DNA, including pathogen-derived DNA or self-DNA from genomic DNA damage, and then synthesizes cGAMP [2,4–8]. cGAMP binds to the dimeric STING located in the endoplasmic reticulum (ER) and that induces STING oligomerization. STING then translocates from ER to the Golgi apparatus, where it activates TANK-binding kinase 1 (TBK1) and interferon regulatory factor 3 (IRF3), leading to the production of type I interferons (IFNs) [3,4,6,7,9–14]. Optimal activation of STING is essential for initiating a robust innate immune response against microbial pathogens and maintaining immune homeostasis. Moreover, emerging evidence has shown that aberrant STING activity leads to autoimmune and autoinflammatory diseases [6,15,16]. Therefore, STING activity should be tightly controlled to facilitate the elimination of pathogens and avoid autoimmune disorders.

Selenium (Se), as an essential trace element, is important for physiological processes and its dysregulation has been implicated in various infectious or noninfectious contexts [17–20]. Viruses hijack host Se supply to achieve immune escape and enhance viral replication and pathogenicity [18–20]. The biological function of Se is mainly through selenoproteins, which incorporate Se in the form of selenocysteine [17,18,21]. Most selenoproteins are soluble proteins located in the cytoplasm and regulate redox pathways to protect against oxidative stress [18,21]. Previously, we reported that the cytoplasmic selenoprotein glutathione peroxidase 4 (GPX4) inhibits lipid peroxidation and maintains cellular redox homeostasis, thereby facilitating antiviral innate immunity by promoting STING trafficking to the Golgi complex and attenuating the immune escape of viruses [22]. Eight selenoproteins are located in ER, while the functions of most of these selenoproteins remain poorly studied [23]. SELENOK is one of the ER transmembrane protein with 94 amino acid residues for humans and mice and contains a highly disordered region in its cytosolic region, which serves as a nonenzymatic structural element to mediate the interaction with other proteins [24]. It has been reported that SELENOK is expressed at relatively high levels in immune cells and its role in innate immunity may through its binding to DHHC6 to promote palmitoylation, particularly palmitoylation and stabilization of IP3 receptor is necessary for calcium flux [24–26]. However, the potential roles of SELENOK on STING activity and antiviral responses remain unclear.

In this study, we screened the functions of ER-located transmembrane selenoproteins in STING activation and found that SELENOK was required for STING activation. SELENOK interacts with STING in the ER and promotes its oligomerization, thereby licensing STING trafficking to the Golgi complex. *Selenok* deficiency suppresses STING-dependent antiviral innate response and facilitates viral replication. Our results shed light on how ER-located transmembrane selenoproteins regulate innate immune responses and suggest SELENOK as a prime target for intervention in aberrant STING activation-caused diseases.

## Results

### SELENOK is indispensable for cGAS-STING pathway

To investigate the potential regulatory roles of ER-located transmembrane selenoproteins on STING-dependent antiviral immunity, we performed an unbiased screening by constructing gene knockdown models using small interfering RNAs (siRNAs) of each ER-located transmembrane selenoproteins in Herpes simplex virus-1- (HSV-1, a DNA virus recognized by cGAS)-infected mouse primary peritoneal macrophages (PMs) (S1 Fig). *Selenok* knockdown markedly attenuated HSV-1 infection-induced *Ifnb* mRNA expression, but the knockdown of other selenoproteins, including iodothyronine deiodinase 2(DIO2), selenoprotein F(SELENOF), Selenoprotein M(SELENOM), selenoprotein N(SELENON), selenoprotein S(SELENOS), selenoprotein T(SELENOT), selenoprotein I(SELENOI), did not (S1 Fig).

**Fig 1:**
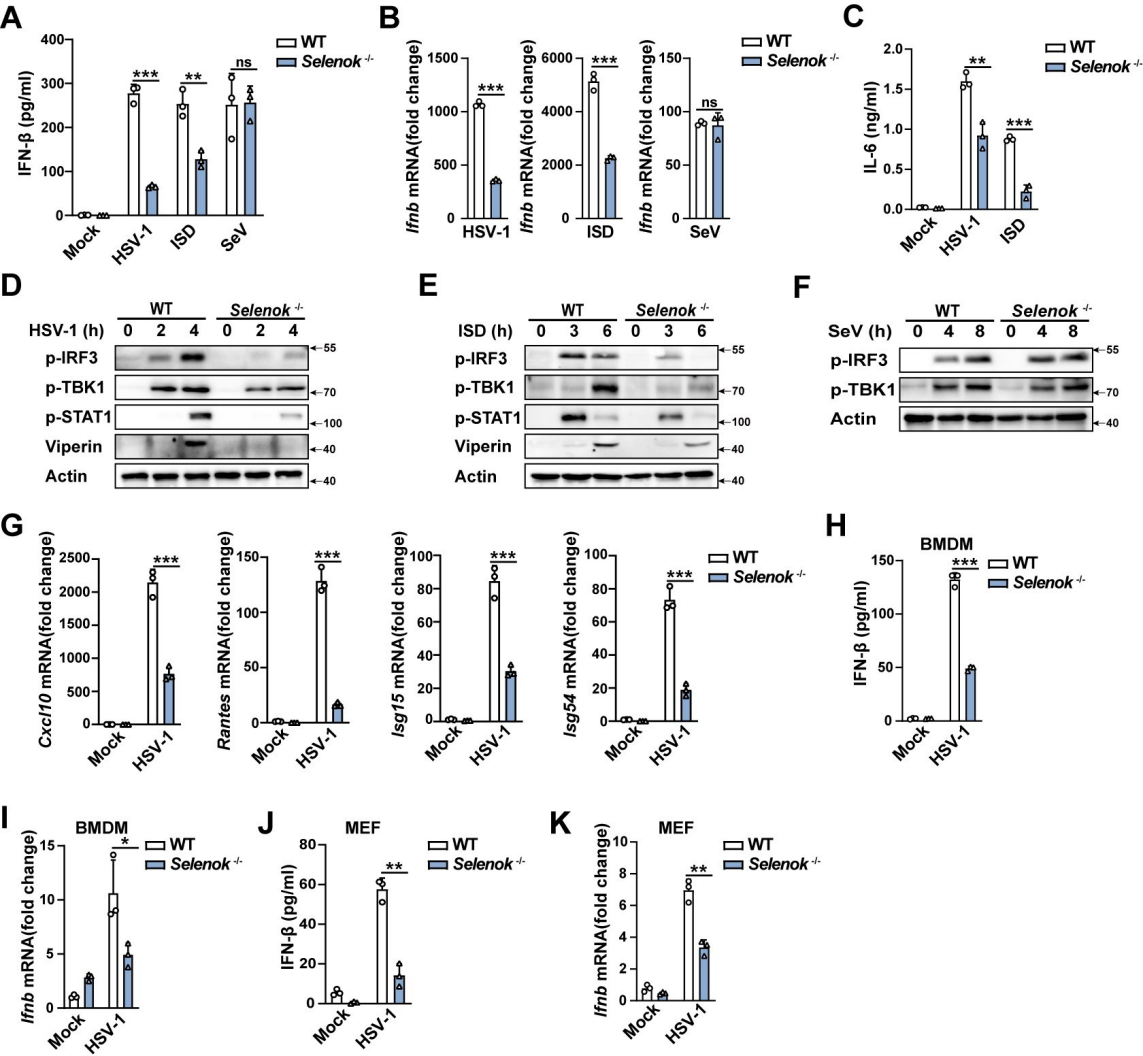
*Selenok* deficiency specifically attenuates cGAS-STING signaling. **(A)** ELISA analysis of IFN-β secretion in PMs following stimulation with HSV-1, ISD, and SeV. **(B)** Real-time PCR analysis of *Ifnb* mRNA levels in PMs following stimulation with HSV-1, ISD, and SeV. **(C)** ELISA analysis of IL-6 secretion in PMs following stimulation with HSV-1 and ISD. **(D-F)** Immunoblot analysis of p-IRF3, p-TBK1, p-STAT1,and Viperin expression in PMs following stimulation with **(D)** HSV-1 or **(E)** ISD or **(F)** SeV. **(G)** Real-time PCR analysis of *Cxcl10*, *Rantes*, *Isg15*, and *Isg54* mRNA levels in PMs following stimulation with HSV-1. **(H)** ELISA analysis of IFN-β secretion in BMDMs following stimulation with HSV-1. **(I)** Real-time PCR analysis of *Ifnb* mRNA levels in BMDMs following stimulation with HSV-1. **(J)** ELISA analysis of IFN-β secretion in MEFs following stimulation with HSV-1. **(K)** Real-time PCR analysis of *Ifnb* mRNA levels in MEFs following stimulation with HSV-1. Statistical significance was determined by unpaired two-sided multiple Student’s t-tests in A-C and G-K. Data represent mean ± standard deviation (SD) or one representative image from three independent experiments. ns: not significant, *P: < 0.05, **P: < 0.01, ***P: < 0.001.

**Fig 2:**
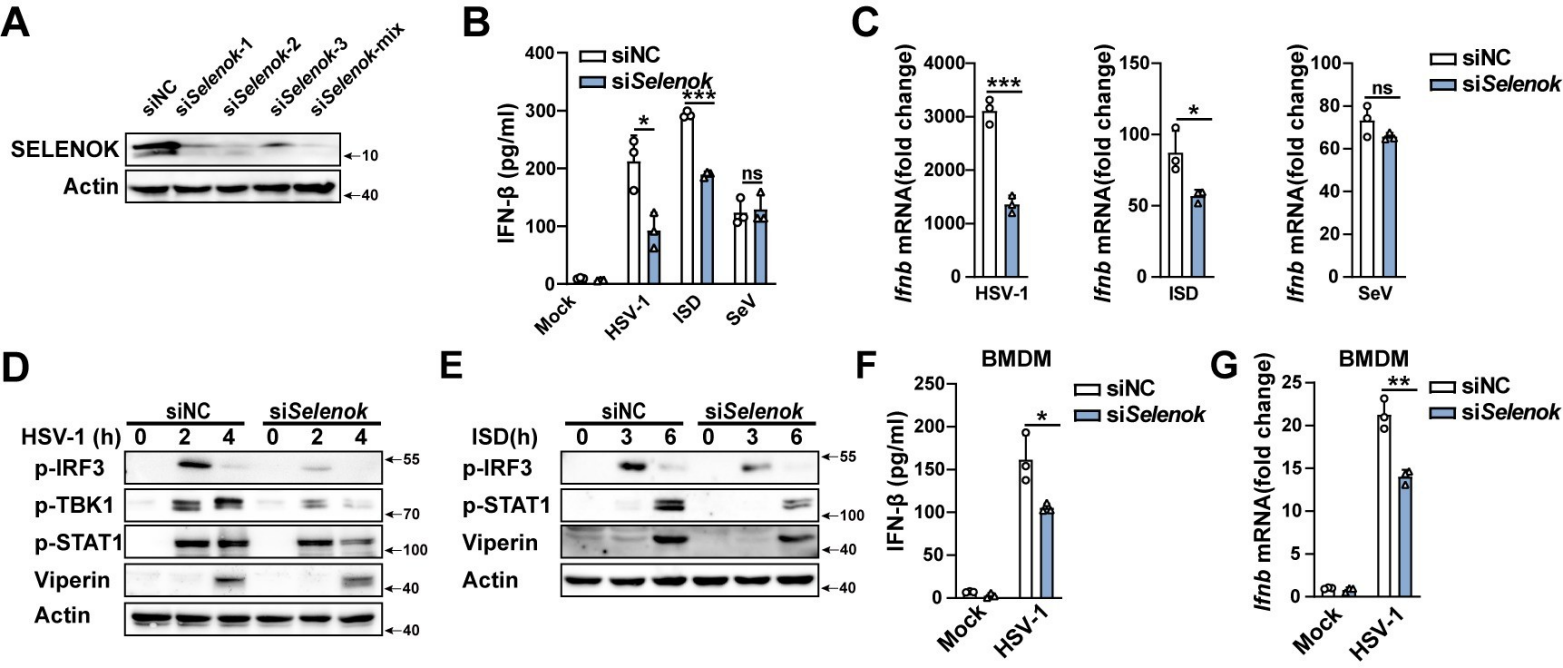
*Selenok* knockdown specifically inhibits cGAS-STING signaling. **(A)** Immunoblot analysis of SELENOK expression in PMs transfected with siRNA. **(B)** ELISA analysis of IFN-β secretion in PMs stimulated with HSV-1, ISD, and SeV after transfection with siRNA. **(C)** Real-time PCR analysis of *Ifnb* mRNA levels in PMs stimulated with HSV-1, ISD, and SeV after transfection with siRNA. **(D)** Immunoblot analysis of p-IRF3, p-TBK1, p-STAT1, and Viperin expression in PMs stimulated with HSV-1 after transfection with siRNA. **(E)** Immunoblot analysis of p-IRF3, p-STAT1, and Viperin expression in PMs stimulated with ISD after transfection with siRNA. **(F)** ELISA analysis of IFN-β secretion in BMDMs stimulated with HSV-1. **(G)** Real-time PCR analysis of *Ifnb* mRNA levels in BMDMs stimulated with HSV-1. Statistical significance was determined by unpaired two-sided multiple Student’s t-tests in B-C and F-G. Data represent mean ± standard deviation (SD) or one representative image from three independent experiments. ns: not significant, *: P < 0.05, **: P < 0.05, ***P: < 0.001. siNC, control siRNA; si*Selenok*-1,2,3, and mix (S1 Table).

We searched the tissue spectrum of mice SELENOK from BioGPS(#1423225) and found that SELENOK was highly expressed in most tissues and immune cells, particularly PMs and bone marrow derived macrophages (BMDMs) (S2A and S2B Fig).To further evaluate the role of SELENOK in cGAS-STING pathway activation, we generated *Selenok*-deficient (*Selenok*^-/-^) mice (S2C–S2G Fig). We compared the cell viability of PMs from WT and *Selenok-*deficient mice in the resting state and found there was no significant difference between these two kinds of PMs (S2H Fig). Moreover, we compared the growth, development, and movement of WT and *Selenok*-deficient mice, and found no significant difference (S2I Fig). The blood routine analysis showed that the proportion of lymphocytes, monocytes, and neutrophils in the immune cells of the two kinds of mice was not significantly different (S2J Fig). These results suggested that *Selenok*-deficient mice exhibit the same developmental or immunological phenotype compared to WT mice in physiological status. Compared to Wild-Type(WT) mice, HSV-1- and interferon-stimulating DNA- (ISD, which can be recognized by cGAS)-induced IFN-β and interleukin (IL)-6 secretion and mRNA expression of *Ifnb* were decreased in *Selenok*-deficient mice PMs (Fig 1A–1C). However, Sendai virus (SeV, an RNA virus recognized by RIG-I)-induced IFN-β secretion and *Ifnb* mRNA expression were not significantly differed in PMs between *Selenok*-deficient mice and WT mice (Fig 1A and 1B). CpG oligonucleotides (CpG ODNs, a kind of TLR9 agonist)-induced IFN-β and IL-6 secretion were similar in PMs and BMDMs from WT or *Selenok*-deficient mice (S3A–S3C Fig). Phosphorylation of TBK1 and IRF3 is crucial for the production of type I IFNs after cGAS-STING activation, and then IFNs activate the JAK-STAT pathway and promote the expression of interferon-stimulated genes (ISGs) to facilitate an antiviral state [14]. HSV-1- and ISD-induced phosphorylation of TBK1 and IRF3 greatly attenuated in *Selenok*-deficient PMs, but the SeV-induced did not (Fig 1D–1F). Consequently, HSV-1 and ISD-induced phosphorylation of STAT1 and the expression of Viperin (a type of ISG) were attenuated in *Selenok*-deficient mice PMs (Fig 1D and 1E). Similarly, HSV-1-induced *Cxcl10*, *Rantes*, *Isg15*, and *Isg54* mRNA expression was suppressed in *Selenok*-deficient mice PMs (Fig 1G). In addition, HSV-1-induced IFN-β secretion and mRNA expression of *Ifnb* were also decreased in *Selenok*-deficient mice BMDMs and mouse embryonic fibroblasts (MEFs) (Fig 1H–1K). To further confirm the regulatory role of SELENOK, endogenous SELENOK expression was knocked down in PMs (Fig 2A), and this selectively inhibited HSV-1- and ISD-induced instead of SeV-induced IFN-β secretion and *Ifnb* mRNA expression (Fig 2B and 2C). The phosphorylation of IRF3, TBK1, STAT1, and Viperin (Fig 2D and 2E) were also greatly decreased in *Selenok*-knockdown PMs. In addition, HSV-1-induced IFN-β secretion and mRNA expression of *Ifnb* were also decreased in *Selenok*-knockdown BMDMs (Fig 2F and 2G). Taken together, these results indicated that SELENOK selectively facilitates the cGAS-STING pathway, with no effect on the RIG-I- and TLR-9-dependent pathways.

### SELENOK enhances host defenses against HSV-1

**Fig 3:**
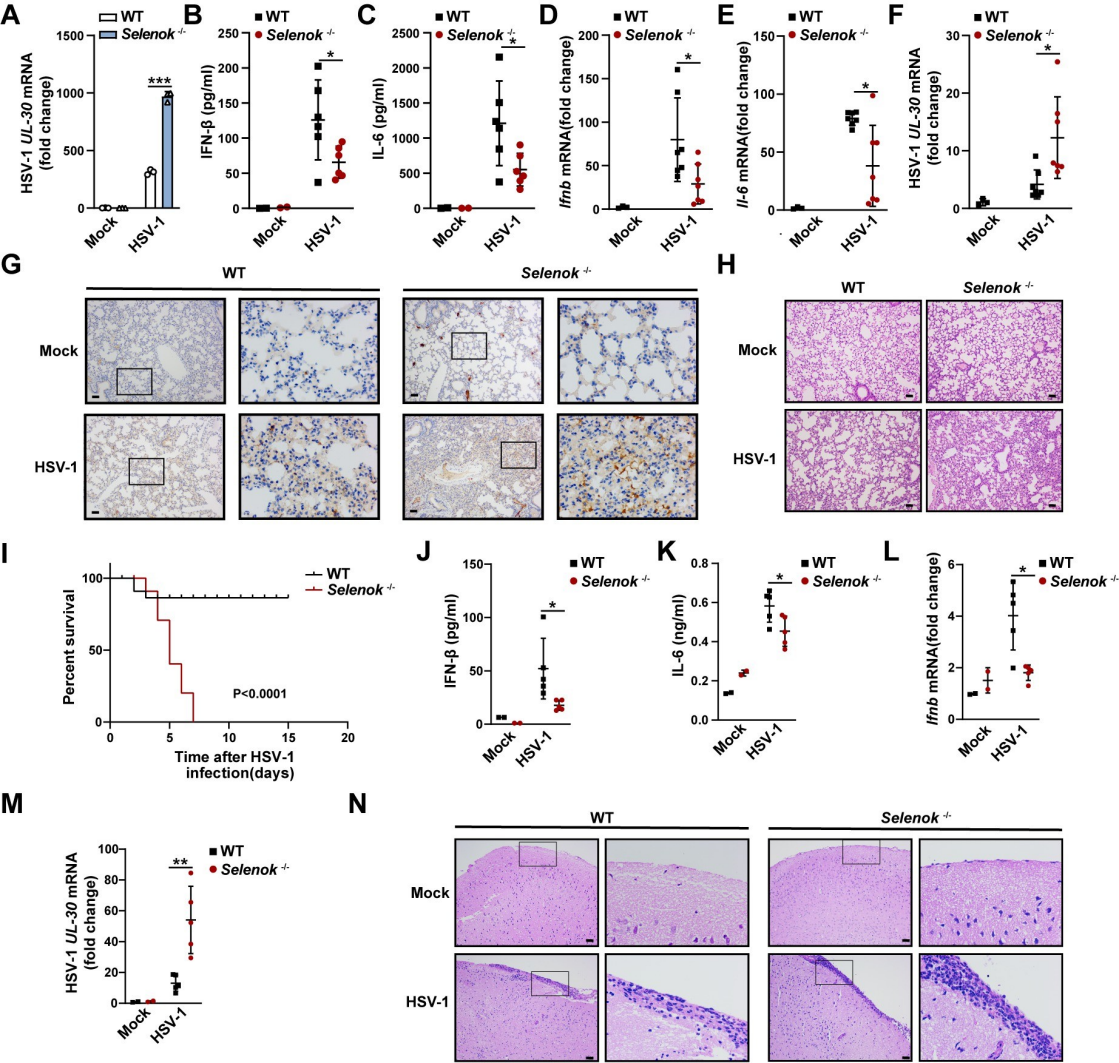
*Selenok* deficiency inhibits innate immune responses against HSV-1 *in vivo*. **(A)**Real-time PCR analysis of HSV-1 *UL30* mRNA levels in PMs stimulated with HSV-1. **(B-F)** Mice were infected with HSV-1 for 8h by i.p. injection. **(B and C)** Serum levels of IFN-β and IL-6 analyzed by ELISA (mock, n = 2; HSV-1, n = 6). **(D and E)** Real-time PCR analysis of *Ifnb* and *Il-6* mRNA levels in spleen homogenate (mock, n = 3; HSV-1, n = 7). **(F)**HSV-1 viral burden determined by real-time PCR analysis of HSV-1 *UL30* mRNA levels in spleen homogenates (mock, n = 3; HSV-1, n = 7). **(G and H)** Mice were infected with HSV-1 for 36h by i.p. injection. **(G)** HSV-1 viral burden was determined by ICP-5 immunohistochemical staining of lung slices. Scale bar, 50μm. **(H)** Hematoxylin and eosin staining of the lung slices. Scale bar, 50μm. **(I)** Kaplan–Meier method was used to evaluate survival curves (n = 15 per condition). **(J-L and N)** Mice were infected with HSV-1 for 12h by intracerebral injection. **(J and K)** Serum levels of IFN-β and IL-6 were analyzed by ELISA (mock, n = 2; HSV-1, n = 5). **(L)** Real-time PCR analysis of *Ifnb* mRNA levels in brain homogenate (mock, n = 2; HSV-1, n = 5). **(M)** Mice were infected with HSV-1 for 40h by intracerebral injection. HSV-1 viral burden determined by real-time PCR analysis of HSV-1 *UL30* mRNA levels in brain homogenates (mock, n = 2; HSV-1, n = 5). **(N)** Hematoxylin and eosin staining of the brain slices. Scale bar, 50μm. Statistical significance was determined by unpaired two-sided multiple Student’s t-tests in A-F and J-M. Data represent mean ± standard deviation (SD) or one representative image from three independent experiments. Graph I was determined by the log-rank Mantel-Cox test. *: P < 0.05, **: P < 0.05, ***P: < 0.001.

Type I IFNs and ISGs are crucial for reducing viral replication and pathogenicity. The replication of HSV-1 was promoted in *Selenok*-deficient mice PMs (Fig 3A), consistent with their inhibitory effects on IFN-β and ISGs expression. Next, we investigated the physiological and pathological relevance of the regulatory effects of SELENOK on host defense in the context of HSV-1 infection *in vivo*. Following HSV-1 infection, *Selenok*-deficient mice had notably less IFN-β and IL-6 in their sera than the WT mice (Fig 3B and 3C). The mRNA expression of *Ifnb* and *Il-6* in the spleen also decreased in *Selenok*-deficient mice (Fig 3D and 3E). In agreement with this, HSV-1 viral burden was increased in the spleen (Fig 3F) and lung (Fig 3G) of *Selenok*-deficient mice compared with that in controls. We also observed increased infiltration of inflammatory cells in the lungs of *Selenok*-deficient mice (Fig 3H). Moreover, *the Selenok*-deficient mice were more susceptible to HSV-1 infection (Fig 3I). The most common neurological disease caused by HSV-1 virus is herpes simplex encephalitis, so we established a model of herpes simplex encephalitis by intracerebral injection, and found that the results were consistent with the results of intraperitoneal injection of the virus. In *Selenok*-deficient mice, the serum secretion of IFN-β and IL-6 decreased (Fig 3J and 3K), and the expression of *Ifnb* mRNA in brain tissue decreased (Fig 3L). Meanwhile, HSV-1 viral burden was also increased in the brain of *Selenok*-deficient mice (Fig 3M). In agreement with this, more serious meningeal edema and inflammatory infiltration in brain tissue of *Selenok*-deficient mice compared with that in controls (Fig 3N). Therefore, these results suggested that *Selenok* deficiency inhibits innate immune responses against HSV-1 *in vivo*.

### SELENOK targets STING

**Fig 4:**
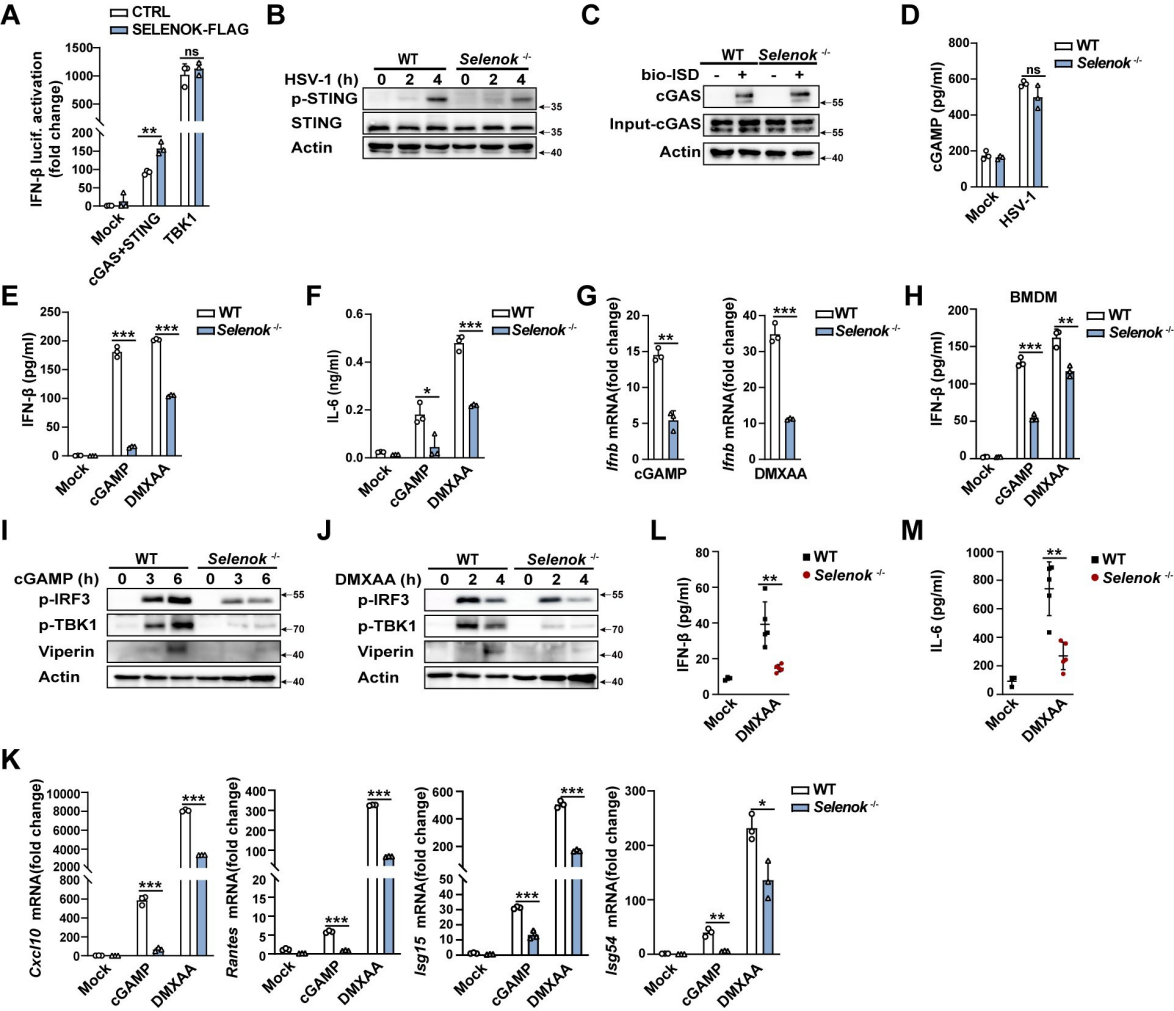
*Selenok* deficiency inhibits STING activation. **(A)** Luciferase activity assays of IFN-β activation in HEK 293T cells transfected with control vector or SELENOK, followed by transfection with control vector (Mock), cGAS plus STING, and TBK1. **(B)** Immunoblot analysis of phosphorylated and total STING in PMs stimulated with HSV-1. **(C)** In vitro pull-down of cGAS and ISD. **(D)** ELISA analysis of cGAMP secretion in PMs stimulated with HSV-1. **(E)** ELISA analysis of IFN-β secretion in PMs following stimulation with cGAMP and DMXAA. **(F)** ELISA analysis of IL-6 secretion in PMs following stimulation with cGAMP and DMXAA. **(G)**Real-time PCR analysis of *Ifnb* mRNA levels in PMs stimulated by cGAMP and DMXAA. **(H)** ELISA analysis of IFN-β secretion in BMDMs following stimulation with cGAMP and DMXAA. **(I and J)** Immunoblot analysis of p-IRF3, p-TBK1, and Viperin expression with **(I)** cGAMP stimulation or **(J)** DMXAA stimulation. **(K)** Real-time PCR analysis of *Cxcl10*, *Rantes*, *Isg15*, and *Isg54* mRNA levels in PMs following stimulation with cGAMP and DMXAA. **(L–M)** Mice were stimulated by DMXAA for 6 h by i.p. injection. **(L)** Serum levels of IFN-β were analyzed using ELISA (mock, n = 3; DMXAA, n = 5). **(M)** Serum levels of IL-6 were analyzed using ELISA (mock, n = 3; DMXAA, n = 5). Statistical significance was determined by unpaired two-sided multiple Student’s t-tests in A, D-H, and K-M. Data represent mean ± standard deviation (SD) or one representative image from three independent experiments. ns: not significant. *: P < 0.05, **P: < 0.01, ***P: < 0.001.

To clarify the mechanism by which *Selenok* deficiency attenuates cGAS-STING signaling, we first performed luciferase reporter assays and found that cGAS and STING, rather than TBK1, could significantly promote IFN-β production (Fig 4A), suggesting that SELENOK may target cGAS or STING. STING phosphorylation was attenuated in *Selenok*-deficient PMs following stimulation with HSV-1 (Fig 4B). However, *Selenok* deficiency did not affect the binding of cGAS to ISD (Fig 4C). cGAMP production during HSV-1 infection was not significantly differed in PMs of *Selenok*-deficient mice and WT mice (Fig 4D). Thus, we speculated that SELENOK might affect the activation of the cGAS-STING pathway by targeting STING. cGAMP is a natural ligand of STING, and 5,6-dimethylxanthenone-4-acetic acid (DMXAA) is a STING agonist. The secretion of IFN-β and IL-6 as well as the mRNA expression of *Ifnb* following stimulation with cGAMP and DMXAA were inhibited in PMs of *Selenok*-deficient mice (Fig 4E–4G). And the IFN-β secretion stimulated by cGAMP and DMXAA was also decreased in BMDMs of *Selenok*-deficient mice (Fig 4H). Concordantly, the activation of IRF3, TBK1, Viperin, and mRNA expression of some ISGs (*Cxcl10*, *Rantes*, *Isg15*, and *Isg54*) were also attenuated (Fig 4I–4K). Consistent with these observations, IFN-β secretion and mRNA expression of *Ifnb* stimulated by cGAMP or DMXAA were greatly decreased in *Selenok*-knockdown PMs (S4A and S4B Fig). The activation of IRF3, TBK1, STAT1, and Viperin stimulated by cGAMP or DMXAA was also attenuated in *Selenok*-knockdown PMs (S4C and S4D Fig). In an animal model of STING activation, compared to WT mice, the secretion of IFN-β and IL-6 in sera from *Selenok*-deficient mice was significantly decreased (Fig 4L and 4M). Collectively, these data indicate that SELENOK targets STING but not cGAS.

### SELENOK interacts with STING and enhances its oligomerization

**Fig 5:**
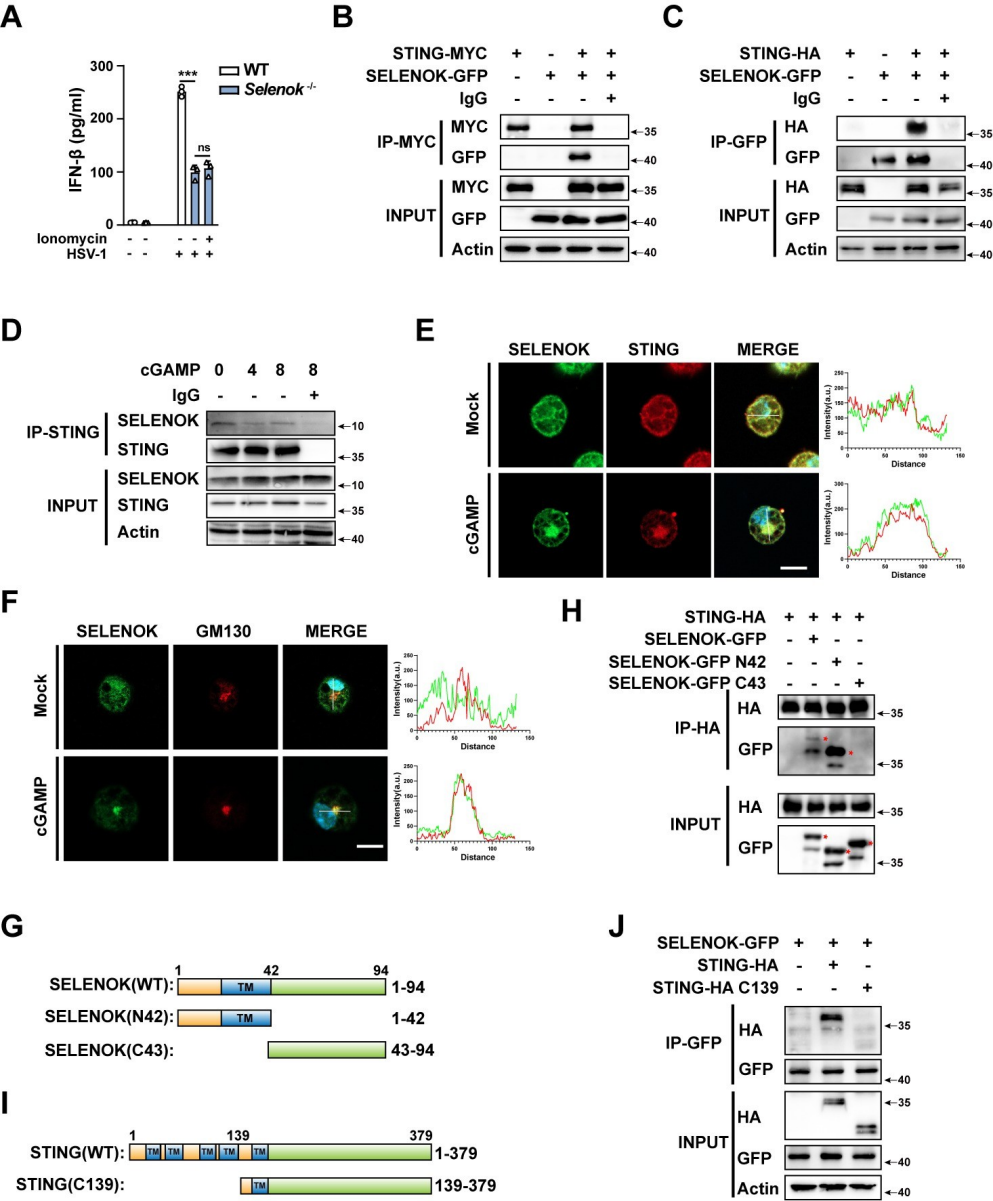
SELENOK interacts with STING. **(A)** ELISA analysis of IFN-β secretion in PMs from WT and *Selenok*-deficient mice stimulated with HSV-1 or ionomycin plus HSV-1. **(B)** Immunoblots of MYC immunoprecipitation in HEK 293T cells transfected with STING-MYC and the control vector or SELENOK-GFP plasmids. **(C)** Immunoblots of GFP immunoprecipitation in HEK 293T cells transfected with SELENOK-GFP and the control vector or STING-HA plasmids. **(D)** Immunoprecipitation using a STING-specific antibody in PMs stimulated with cGAMP. **(E)** Immunofluorescence analysis of PMs stimulated by cGAMP or not using anti-SELENOK (green) and anti-STING (red) antibodies and DAPI nuclear staining. Scale bar,10μm. The intensity profiles of each line were quantified by Image J software. **(F)** Immunofluorescence analysis of PMs stimulated by cGAMP or not using anti-SELENOK (green) and anti-GM130 (red) antibodies and DAPI nuclear staining. Scale bar,10μm. The intensity profiles of each line were quantified by Image J software. **(G)** Schematic of the SELENOK and its truncated body. **(H)** HA immunoprecipitation in HEK 293T cells transfected with STING-HA and the control vector, SELENOK-GFP, SELENOK-GFP N42, or SELENOK-GFP C43 plasmids. The specific bands were marked by asterisks. **(I)** Schematic of the STING and its truncated body. **(J)** GFP immunoprecipitation in HEK 293T cells transfected with SELENOK-GFP and the control vector, STING-HA, STING-HA C139. Statistical significance was determined by unpaired two-sided multiple Student’s t-tests in A. Data represent mean ± standard deviation (SD) or one representative image from three independent experiments. ns: not significant; ***P: < 0.001.

*Selenok*-deficient macrophages have also been reported to exhibit decreased receptor-mediated calcium flux [27]. To understand how *Selenok* deficiency attenuates STING activation, we first used ionomycin (a kind of Ca^2+^ ionophore that could open Ca^2+^ channels and intracellular stores) to treat *Selenok*-deficient macrophages and found that ionomycin treatment cannot reverse the decrease of IFN-β secretion caused by *Selenok* deficiency (Fig 5A), suggesting that receptor-mediated calcium flux does not affect *Selenok*-deficient-mediate STING activation. SELENOK protein has a disordered region and can act as a scaffold for interaction with other proteins [24,26,28,29]. Next, we examined the interaction between SELENOK and STING by co-transfecting SELENOK and STING in the human embryonic kidney (HEK) 293T cells, and we found that SELENOK co-precipitated with STING (Fig 5B and 5C). SELENOK also interacted with STING in the mouse PMs (Fig 5D). Confocal analysis demonstrated colocalization between SELENOK and STING in the PMs (Fig 5E). In addition, the confocal analysis demonstrated colocalization between SELENOK and GM130, which means SELENOK co-traffics with STING to Golgi upon its activation (Fig 5F). In humans, SELENOK is composed of 94 amino acids [24]. The N-terminal half (1–42) of SELENOK is located in the ER, and the C-terminal half (43–94) is in the cytoplasm (Fig 5G). To identify the domain of SELENOK that is responsible for the interaction with STING, a number of SELENOK-truncated mutants were constructed. STING co-precipitated with the N-terminal half of SELENOK in the ER, but not the C-terminal half (Fig 5H). STING is also a transmembrane protein in the endoplasmic reticulum, and its positions 1–139 are multiple transmembrane domains (Fig 5I). The binding ability of STING and SELENOK is greatly reduced between wild-type STING and C139 of STING (deleted multiple transmembrane domains of STING) (Fig 5J). Therefore, we suspect that the interaction between SELENOK and STING in the endoplasmic reticulum was conducive to STING oligomerization.

**Fig 6:**
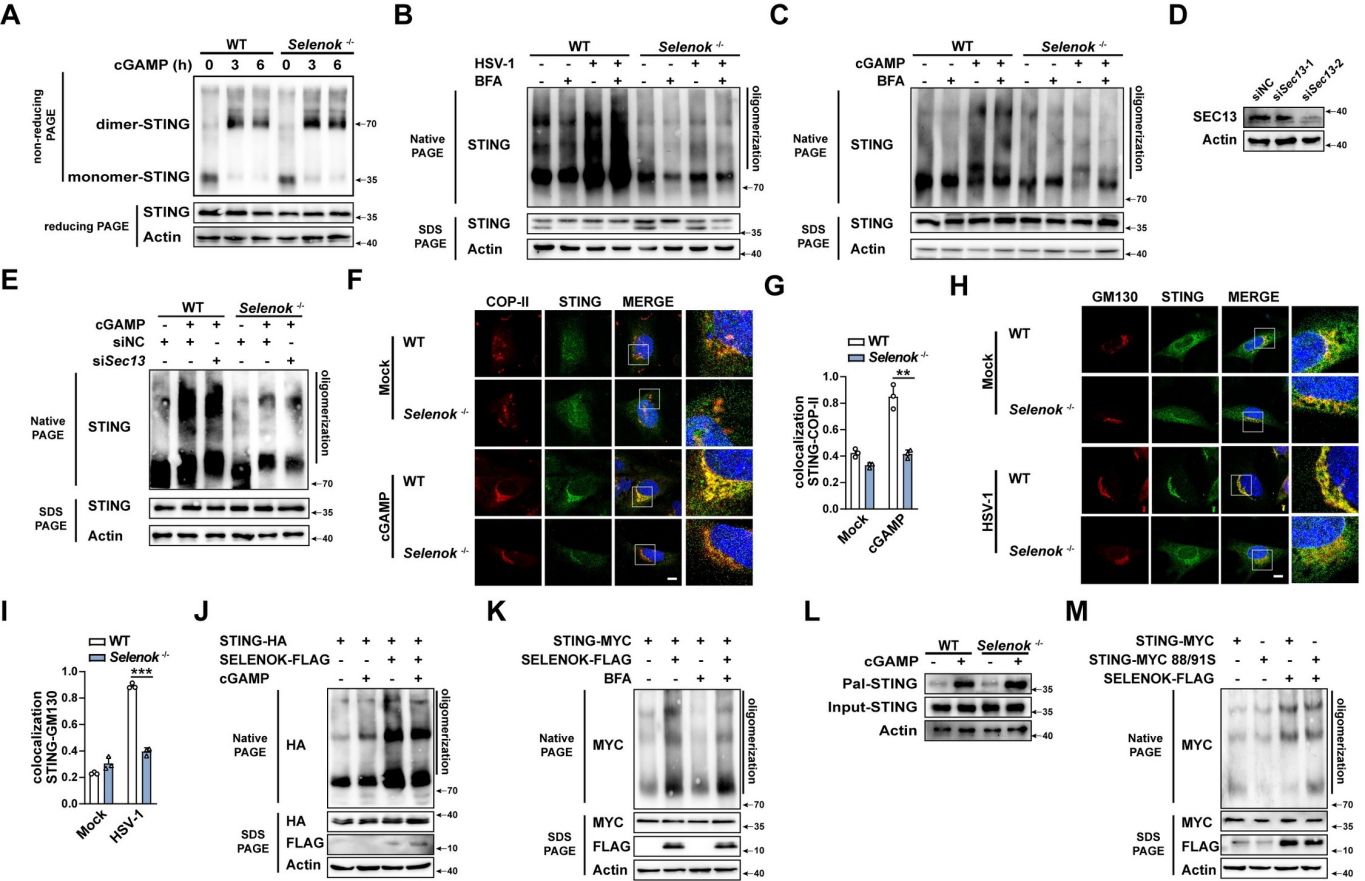
SELENOK enhances the oligomerization of STING. **(A)** Immunoblot analysis of STING dimerization in PMs stimulated by cGAMP. **(B)** Immunoblot analysis of oligomerization of STING in PMs stimulated by BFA, HSV-1, or BFA plus HSV-1. **(C)** Immunoblot analysis of oligomerization of STING in PMs stimulated by BFA, cGAMP, or BFA plus cGAMP. **(D)** Immunoblot analysis of SEC13 expression in PMs transfected with siRNA (siNC, si*Sec13*-1 and si*Sec13*-2). **(E)** Immunoblot analysis of oligomerization of STING in PMs stimulated by cGAMP after transfection with siRNA (siNC, si*Sec13*-2). **(F)** Immunofluorescence analysis of MEF stimulated with cGAMP using anti-STING (green) and anti-COP-II (red) antibodies and DAPI nuclear staining. Scale bar,10μm. **(G)** Colocalization analysis of STING and COP-II by Manders’ Colocalization Coefficients (MCC). **(H)** Immunofluorescence analysis of MEF stimulated with HSV-1 using anti-STING (green) and anti-GM130 (red) antibodies and DAPI nuclear staining. Scale bar,10μm. **(I)** Colocalization analysis of STING and GM130 by Manders’ Colocalization Coefficients (MCC). **(J)** Immunoblot analysis of STING oligomerization in HEK 293T cells transfected with STING-HA and control vector plasmids or SELENOK-FLAG, followed by stimulation with cGAMP. **(K)** Immunoblot analysis of STING oligomerization in HEK 293T cells transfected with STING-MYC, the control vector or SELENOK-FLAG, and then stimulated by BFA or not. **(L)** Immunoblot analysis of STING palmitoylation in PMs stimulated by cGAMP. **(M)** Immunoblot analysis of STING oligomerization in HEK 293T cells transfected with STING-MYC, STING-MYC C88/91S, the control vector, and SELENOK-FLAG. Statistical significance was determined by unpaired two-sided multiple Student’s t-tests in G and I. Data represent mean ± standard deviation (SD) or one representative image from three independent experiments. **P: < 0.01, ***P: < 0.001. siNC, control siRNA; si*Sec13*-1; si*Sec13*-2 (S1 Table).

Under resting conditions, STING is anchored to the ER membrane in the form of a homodimer, and the binding of cGAMP induces STING dimerization and oligomerization to license its translocation from the ER to the Golgi complex via coat protein complex II (COP-II) [3,10]. The dimerization of STING was not affected by *Selenok* deficiency (Fig 6A). Brefeldin A (BFA) is a specific inhibitor that blocks the transport of protein from ER to Golgi [30,31]. Previous studies found that treatment with BFA inhibits the translocation of STING from the ER to the Golgi apparatus but does not affect the oligomerization of STING, suggesting that the oligomerization of STING takes place in the ER before STING trafficking [3,32]. Therefore, we treated with BFA in WT or *Selenok*-deficient PMs to confirm whether the oligomerization of STING is affected by SELENOK in ER. Our results showed that *Selenok*-deficient inhibited HSV-1 infection and cGAMP-induced STING oligomerization with both control solvent or BFA treatment (Fig 6B and 6C; 3rd lane compared to 7th lane and 4th lane compared to 8th lane). SEC13 is an important component of COP-II, which has been reported to play a significant role in STING translocation to Golgi [33]. Thus, we used small interfering RNA (siRNA) knocking down Sec13 to destroy the vesicular transport of COP-II (Fig 6D) and then detected the oligomerization in WT and *Selenok*-deficient PMs. The result is consistent with the BFA treatment (Fig 6E), which confirmed that SELENOK promoted STING oligomerization before its trafficking to the Golgi apparatus. Concordantly, the translocation of STING to COP vesicle or Golgi was decreased in *Selenok*-deficient MEFs compared to WT MEFs (Fig 6F–6I). Next, we confirmed the function of SELENOK in the regulation of STING oligomerization. In HEK 293T cells, we transfected the STING plasmid and found that SELENOK enhanced cGAMP-induced STING oligomerization (Fig 6J). Interestingly, SELENOK overexpression directly enhanced STING oligomerization in HEK293T cells without cGAMP and could not be inhibited by BFA (Fig 6K). Furthermore, our experiment found that *Selenok* deficiency does not decrease the palmitoylation of STING (Fig 6L). Then we constructed STING palmitoylation site mutant plasmid (C88S and C91S, in which two Cys (C)-to-Ser (S) point mutations were introduced) to detect whether the palmitoylation of STING is involved in SELENOK-induced STING oligomerization. The results showed that the C88S and C91S mutants do not affect SELENOK-induced STING oligomerization (Fig 6M). The palmitoylation of STING is important for its trafficking and oligomerization [13], but it is unknown whether the palmitoylation of STING occurs in the endoplasmic reticulum or the Golgi apparatus and whether the trafficking of STING directly affects its palmitoylation. Thus, our results indicated the function of SELENOK in STING oligomerization in a palmitoylation-independent manner. Taken together, these data suggest that SELENOK enhances the oligomerization of STING in the ER and licenses its trafficking from the ER to the Golgi complex.

### IFN-β promotes the expression of SELENOK

**Fig 7:**
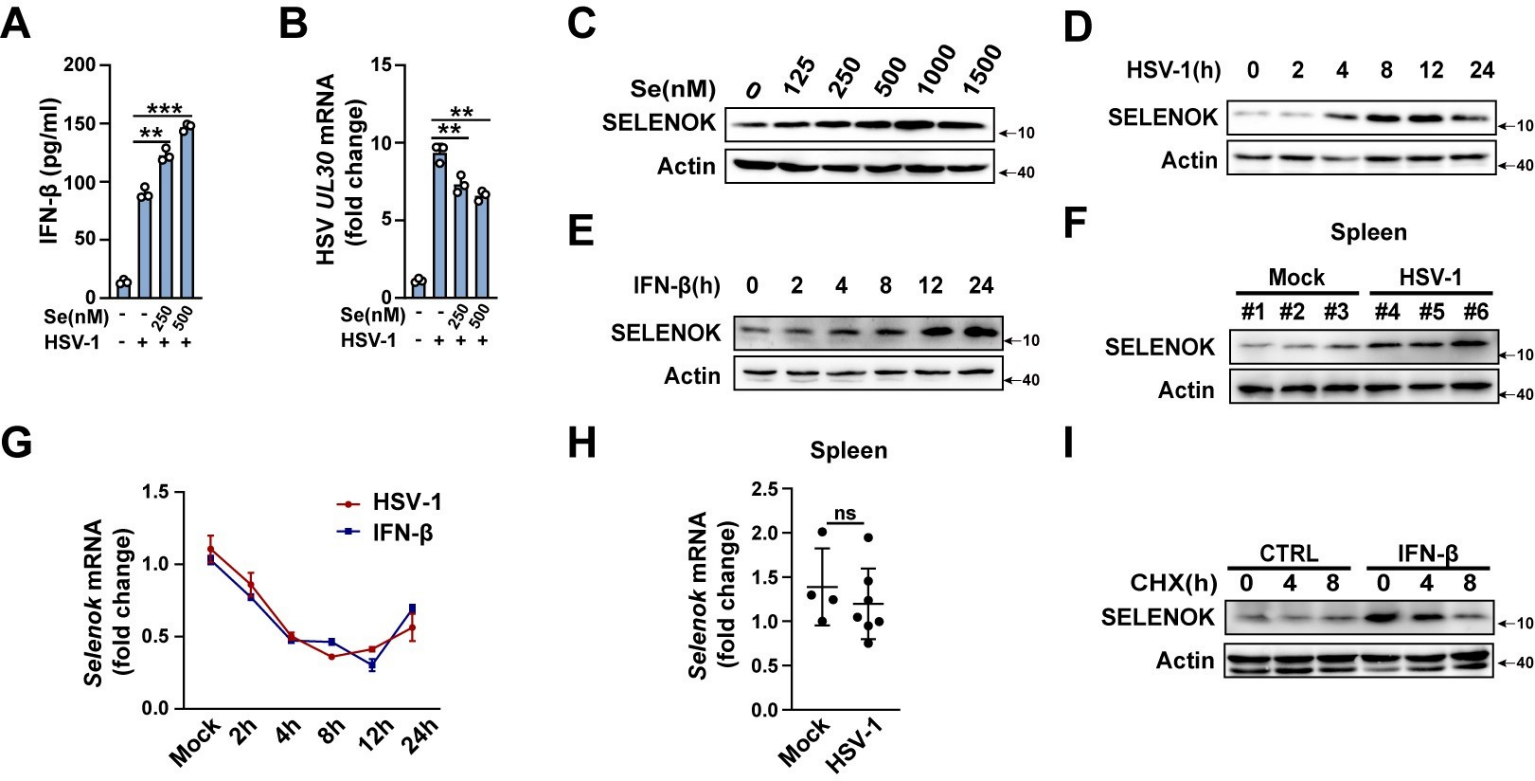
IFN-β promotes the expression of SELENOK. **(A)** ELISA analysis of IFN-β secretion in PMs following stimulation with Se and HSV-1. **(B)** Real-time PCR analysis of HSV-1 *UL30* in PMs following stimulation with Se and HSV-1. **(C)** Immunoblot analysis of SELENOK expression in PMs stimulated by Se with increasing concentration. **(D and E)** Immunoblot analysis of SELENOK expression in PMs with stimulation of HSV-1 and IFN-β over a time gradient. **(F)** Mice were infected with HSV-1 for 12h by i.p. injection. Immunoblot analysis of SELENOK expression in spleen homogenate (mock, n = 3; HSV-1, n = 3). **(G)** Real-time PCR analysis of *Selenok* mRNA levels in PMs with stimulation of HSV-1 and IFN-β over a time gradient. **(H)** Mice were infected with HSV-1 for 12h by i.p. injection. Real-time PCR analysis of *Selenok* mRNA levels in spleen homogenate (mock, n = 4; HSV-1, n = 7). **(I)** Immunoblot analysis of SELENOK expression in PMs stimulated by IFN-β and CHX. Statistical significance was determined by unpaired two-sided multiple Student’s t-tests in A, B, and H. Data represent mean ± standard deviation (SD) or one representative image from three independent experiments. ns: not significant. **P: < 0.01, ***P: < 0.001.

Selenium is important for physiological processes by maintaining the synthesis and expression of selenoproteins [34,35]. The selenium treatment with increased concentration promoted HSV-1 infection-induced IFN-β production and inhibited HSV-1 replication in wild-type PMs (Fig 7A and 7B). Furthermore, selenium treatment enhanced SELENOK protein expression with increased concentration (Fig 7C). Interestingly, HSV-1 and IFN-β stimulation markedly induced SELENOK expression in PMs (Fig 7D and 7E) and *in vivo* (Fig 7F). We further investigated the mechanism by which IFN-β-induced the increase of SELENOK protein expression. Our results showed that IFN-β and HSV-1 stimulated do not enhance the mRNA expression of *Selenok* (Fig 7G and 7H). Moreover, the IFN-β-mediated increase of SELENOK protein expression could be reversed by cycloheximide (CHX, a protein synthesis inhibitor) (Fig 7I), suggesting that IFN-β promoted SELENOK protein synthesis but not the transcription of *Selenok*. Taken together, these data suggest that IFN-β-induced SELENOK expression provides positive feedback to enhance STING-dependent host defense.

## Discussion

The nutritional status of the host is considered to be a key regulator of infectious diseases and involves complex interactions with the immune system [36–41]. Viral infection increases the demand for micronutrients and causes nutritional imbalance in the host, leading to enhanced immune escape and pathogenicity [37–41]. It has been reported that the nutritional status controls the cGAS–STING pathway. The manganese released from membrane-sealed organelles during virus infection enhanced the binding affinity of dsDNA-cGAS and cGAMP-STING and promoted anti-DNA virus innate immune response [38,39]. Zinc enhanced cGAS enzyme activity and stabilized the cGAS-DNA complex by promoting its phase separation [40]. Ca^2+^ sensor stromal interaction molecule 1 (STIM1) is associated with STING and retains it in the ER membrane, limiting its activation [41]. Selenium is an essential dietary trace element associated with adverse clinical outcomes during viral infections, and its biological function is mainly through selenoproteins [17–20]. SELENOK is an ER transmembrane protein and serves as a nonenzymatic structural element that mediates the interaction with other proteins [24,26,28,29]. SELENOK is expressed at relatively high levels in immune cells and is related to inflammation and immune response [25,26,29]. In this study, we demonstrated that SELENOK facilitates the activation of the cGAS-STING pathway by promoting STING oligomerization in the ER, which permits it to translocate from the ER to the Golgi. *Selenok* deficiency facilitated HSV-1 replication and suppressed the STING-triggered type I IFN response. Therefore, SELENOK is required for STING activation and anti-DNA virus responses.

Oligomerization of STING is an essential prerequisite for STING activation [3,9,11,12]. The palmitoylation of STING at two cysteine residues (Cys88 and Cys91) in the Golgi facilitates STING oligomerization and localization to the Golgi [8,13]. Furthermore, sulfated glycosaminoglycans (sGAGs) in the Golgi bind and promote STING oligomerization through luminal, positively charged, polar residues [12]. However, it has been reported that retaining STING in the ER does not block cGAMP-induced STING oligomerization, suggesting that oligomerization events take place in the ER before its localization to the Golgi [3]. SELENOK, unlike most selenoproteins, lacks the CXXU (Cys-XX-Sec) motif, which suggests that it cannot perform biological functions through redox regulation. However, SELENOK contains an intrinsically disordered domain and can bind to other proteins to perform its function or to affect structural changes in chaperone proteins [24]. Our results indicate that SELENOK interacts with STING in the ER in its resting state and enhances STING oligomerization in the ER. SELENOK overexpression directly enhances STING oligomerization without cGAMP. Therefore, the interaction between SELENOK and STING in the ER is important for STING activation.

Selenium and selenoproteins play essential roles in regulating the functions of immune cells and immune responses [17,18,22,42–44]. For example, Se supplementation suppresses Th1 cell differentiation via selenoprotein W (SELENOW)-mediated cellular reactive oxygen species scavenging [42]. Hepatitis C virus (HCV) infection increases the levels of hepatic selenoprotein P (*Selenop*) mRNA, which binds directly to RIG-I and inhibits its activity, resulting in the inhibition of the type I IFN response [43]. Furthermore, Se supplementation inhibits ferroptosis of follicular helper T cells via the selenoprotein GPX4 [44]. Cellular redox homeostasis maintained by GPX4 inhibits STING carbonylation and attenuates the immune escape of HSV-1 [22]. However, the role of ER-located transmembrane selenoproteins in immune response remains largely unknown. In the present study, we showed that virus infection and IFN-β stimulation induce SELENOK expression, which helps achieve fine-tuned regulation of host antiviral responses via STING activation and promotion of virus clearance.

In summary, our study identified SELENOK as an antiviral host factor in the innate immune response and clarified the role of SELENOK in STING activation, that is, the promotion of its oligomerization. The aberrant activation of STING has been linked to several pathological conditions, suggesting that STING is an attractive target for pharmacological modulation. Our results suggest that targeting the control of STING activation by Se-mediated SELENOK expression may be a prime therapeutic strategy for the treatment of STING-associated diseases (S5 Fig).

## Materials and methods

### Ethics statement

All animal experiments were performed in accordance with the National Institutes of Health Guide for the Care and Use of Laboratory Animals and approved by the Scientific Investigation Board of the School of Basic Medical Science, Shandong University, Jinan, Shandong Province, China. The approval number is LL-201602059.

### Mice

C57BL/6N *Selenok*^-/-^ mice (80795) were obtained from Cyagen Biosciences (Guangzhou, China). Mice from both sexes, 6–11 weeks of age were used. All experiments were performed using age and sex matched *Selenok*^*-/-*^ and WT mice.

### Viral infection *in vivo*

All *in vivo* experiments were performed using age and sex matched *Selenok*^*-/-*^ and WT mice. Intraperitoneal injection model: The mouse model of intraperitoneal injection of HSV-1 has been widely used in previous studies [22,45]. Mice at 8 weeks of age were intraperitoneally (i.p) injected with HSV-1 (2×10^7^ p.f.u. per mouse). Serum and spleen were collected 8h after viral infection for ELISA and real-time polymerase chain reaction (PCR), respectively. The mice were euthanized 36h after viral infection to obtain lung tissues. Dissected lung tissues were fixed in 10% phosphate-buffered formalin, embedded in paraffin, and sectioned. Sections were stained with hematoxylin and eosin and examined by light microscopy or with ICP5 and examined for HSV-1 replication. For survival experiments, all mice were injected with equal amounts of HSV-1 and monitored for survival. STING hyperactivation model: DMXAA (23 mg/kg in DMSO) or 100 μl of 50% DMSO in PBS was intraperitoneally injected into mice at 8 weeks of age and serum was collected 6h later for ELISA. Intracerebral injection model: Mice at 8 weeks of age were intracerebrally injected with HSV-1 (3×10^5^p.f.u. per mouse). Serum was collected 12h after viral infection for ELISA. The brain was collected 12h after viral infection for real-time PCR. Dissected brain tissues were fixed in 10% phosphate-buffered formalin, embedded in paraffin, and sectioned. Sections were stained with hematoxylin and eosin and examined by light microscopy. The mice were euthanized 40h after viral infection to examine HSV-1 replication of the brain.

### Reagents and antibodies

cGAMP (tlrl-nacga23-1), DMXAA (tlrl-dmx), ISD (tlrl-isdn), and CpG ODN (tlrl-1826-1) were obtained from InvivoGen. Brefeldin A (BFA), Ionomycin, and cycloheximide (CHX) were purchased from Selleck Chemicals. Se was obtained from Sigma–Aldrich. Palmitic acid probes (C10265) were from Invitrogen. Biotin-alkyne (B171422) was from Aladdin. CCK-8 reagent (HY-K0301) was obtained from MedChemExpress. The sequences of synthetic IFN-stimulating DNA (ISD) were as follows: F5’-TACAGATCTACTAGTGATCTATGACTGATCTGTACTGATCTACA-3’ and R5’-TGTAGATCATGTACAGATCAGTCATAGATCACTAGTAGATCTGTA-3’, which were modified with biotin. Stimulants were used at the following concentrations: ISD, 10μg mL^-1^, cGAMP, 3μg mL^-1^; DMXAA, 150μg mL^-1^; Brefeldin A, 40μM; Ionomycin,1μM; CpG ODNs 2μM; CHX 10μM. SeV was purchased from the China Center for Type Culture Collection. HSV-1 was gifted by X. Cao (Second Military Medical University, Shanghai, China). The STING human recombinant protein (TP308418) was obtained from Origene. Anti-STING (#13647), Anti-p-STING (#72971), anti-p-IRF3 (Ser396; #4947), anti-p-STAT1 (Tyr701; #9167), anti-cGAS (#31659), anti-HA (#3724), anti-streptavidin-HRP (#3999), and anti-rabbit IgG (#2729) antibodies were purchased from Cell Signaling Technology. Anti-SELENOK (ab139949), anti-ICP5 (ab6508), anti-GM130 (ab52649), and anti-p-TBK1 (ab109272) antibodies were purchased from Abcam. Alexa Fluor 633 (A-21071), Alexa Fluor 488 (A-11059), anti-beta-COP-II (PA1-061), and streptavidin (a magnetic bead conjugate) were purchased from Thermo Fisher Scientific. Anti-Myc (M4439) and anti-FLAG (F1804) antibodies were purchased from Sigma-Aldrich. The anti-β-actin (66009-I-Ig), anti-GFP (66002-I-Ig), and anti-SEC13 (15397-1-AP) antibodies were purchased from Proteintech. The anti-mouse IgG (#AC011) was purchased from ABclonal. Protein G agarose (sc-2002) was used for immunoprecipitation and horseradish peroxidase-conjugated secondary antibodies were obtained from Santa Cruz Biotechnology.

### Cells

To obtain primary PMs, 3% Brewer’s thioglycolate was injected (i.p) into C57BL/6N or *Selenok*^-/-^ mice. Three days later, mouse peritoneal exudate cells (PECs) were harvested by irrigation of the peritoneal cavity. PECs were cultured for 2h to remove non-adherent cells, and the remaining adherent monolayer cells were used as PMs. To obtain BMDMs, use a syringe to blow out the bone marrow of the mouse leg bone, and repeatedly blow it into a single-cell suspension. After removing the red blood cells, centrifuge, discard the supernatant, and resuspended with DMEM complete culture medium (penicillin 100U/ml, streptomycin 100U/ml, M-CSF 20ng/ml). Change the solution on the 3rd and 5th days, and then obtain BMDMs after seven days of induction. Human embryonic kidney (HEK) 293T cells were obtained from the American Type Culture Collection. MEFs were generated from 13 to 14 days old fetal mice. The head, limbs, and viscera of the fetal mice were removed, and the remaining embryonic tissues were minced and incubated with 0.25% trypsin-EDTA for 30 min at 37°C. The mixtures were centrifuged and resuspended in DMEM supplemented with 10% FBS. The MEFs were thus obtained and passaged for subsequent experiments. All cells were authenticated by morphology, karyotyping, and PCR-based approaches and checked using the MycoProbe detection kit (R&D Systems) to ensure that there was no mycoplasma contamination. All the cells were cultured at 37°C under 5% CO_2_ in DMEM supplemented with 10% FBS (Gibco), 100U mL^-1^ penicillin, and 100μg mL^-1^ streptomycin.

### Plasmids and transfection

The plasmids SELENOK-GFP, SELENOK-FLAG, SELENOK-GFP N42, SELENOK-GFP C43, and STING-MYC C88/91S were procured from Biosune Biotechnology (Shanghai, China). The IFN-β reporter plasmid and expression plasmids for cGAS, STING, and TBK1 have been described before [22]. The STING-HA C139 mutants were generated using the KOD-Plus-Mutagenesis kit (Toyobo). All plasmids were verified by DNA sequencing. These plasmids were transfected into HEK 293T cells using Lipofectamine 2000 reagent (Invitrogen). The siRNA duplexes were transfected into cells with the INTERFERin reagent (Polyplus-transfection) according to the manufacturer’s protocol. The siRNA sequences are listed in S1 Table. When it came to knocking down the genes with multiple siRNA sequences in S1 Table, the multiple siRNAs were mixed for use.

### Luciferase activity assay

Luciferase activity was measured using the Dual-Luciferase Reporter Assay system according to the manufacturer’s instructions (Promega). Data were normalized for transfection efficiency by dividing firefly luciferase activity by that of Renilla luciferase.

### ELISA

ELISA kits for IFN-β (BioLegend) and IL-6 (DAKEWE) were used to quantify the respective cytokines in the cellular supernatant or serum. Intracellular cGAMP levels were detected using a cGAMP ELISA kit (Cayman).

### Real-time PCR

Total RNA was extracted using the RNAfast200 RNA extraction kit (Fastagen) and reverse-transcribed into cDNA using reverse transcriptase (Vazyme), and real-time PCR analysis was performed using SYBR RT-PCR kits (Vazyme). The primers used for real-time PCR assays are listed in S2 Table.

### Immunoblot analysis

Cells were lysed in RIPA buffer (Pierce, Thermo Scientific) with a protease inhibitor cocktail (Sigma-Aldrich) and phosphatase inhibitor (CWBIO). The samples were centrifuged to remove insoluble precipitates. The protein concentration was measured using the Pierce BCA protein assay kit (Thermo Scientific). The cell lysates were boiled with the loading buffer and separated by SDS-PAGE gel electrophoresis. For non-reducing PAGE, the cell lysates were mixed with loading buffer without boiling. Before SDS-PAGE, the gel was pre-run for 1h. Electrophoresis was performed on ice under low voltage. Then, the proteins were transferred onto nitrocellulose membranes (Millipore) for immunoblot analysis as described previously [22]. For Native PAGE, cells were lysed in lysis buffer (NaCl 0.44g, NP-40 500uL,0.5M Tris-HCl (PH7.5) 5mL,0.5M EDTA 5mL, ddH_2_O 35mL) with a protease inhibitor cocktail (Sigma-Aldrich). The samples were centrifuged to remove insoluble precipitates. The protein concentration was measured using the Pierce BCA Protein Assay Kit (Thermo Scientific). The protein was mixed with loading buffer without SDS and subjected to native gel electrophoresis for immunoblot analysis.

### Immunoprecipitation (IP)

Cells were lysed in IP lysis buffer (50 mM Tris-HCl (pH 7.4), 50 mM EDTA,150 mM NaCl, and 1% NP-40) with a protease inhibitor cocktail (Sigma-Aldrich). The cell lysates were immunoprecipitated with indicated antibodies using Protein A/G PLUS-Agarose (Santa Cruz) overnight at 4°C. The beads were washed five times with IP lysis buffer and boiled in IP buffer containing 1% (w/v) SDS to elute immunoprecipitates.

### *In vitro* pull-down assay

PMs were lysed in lysis buffer (20 mM Tris–HCl, pH 7.5, 0.5% NP-40, 250 mM NaCl, 3 mM EDTA, and 3 mM EGTA) with a protease inhibitor cocktail (Sigma-Aldrich). Lysates were incubated with biotin-ISD at 4°C for 4h and then incubated with streptavidin beads at 4°C for another 2h. The beads were washed five times with wash buffer (20 mM Tris–HCl, pH 7.5, 0.5% NP-40, 10 mM NaCl, 3 mM EDTA, and 3 mM EGTA) and boiled in lysis buffer containing 1% (w/v) SDS. The samples were then analyzed by immunoblotting.

### Immunofluorescence and confocal microscopy

MEFs and PMs were plated on climbing sheets for further treatment. The treated cells were fixed with Immunol Staining Fix Solution (Beyotime) and permeabilized with 0.5% Triton-X 100 in PBS. The climbing sheets were blocked in 3% BSA for 1h, incubated with primary antibodies overnight, and stained with a secondary antibody conjugated to either Alexa Fluor633 (Thermo) or Alexa Fluor 488 (Thermo). Cell nuclei were stained with DAPI (Beyotime). A Zeiss LSM880 confocal laser microscope (Micro Characterization Facility, Shandong University) was used for image capture and analysis.

### Validation of protein palmitoylation by click chemistry

PMs were incubated with 100μM of the palmitic acid probes (Invitrogen C10265) for 4 h at 37°C. Cells were washed twice in PBS, then lysed on ice in 250μl of 50 mM Tris pH 8.0 containing 0.4% SDS and protease inhibitors, and the lysate was incubated with 1 mM CuSO4, 100μM TBTA ligands, 100μM biotin-alkyne and 1 mM tris (2-carboxyethyl) phosphine for 4 h at 25°C. After precipitation, protein bodies were enriched by the addition of streptavidin beads overnight. Samples were washed three times with PBS, and SDS loading buffer was used to elute proteins at 95°C for 20 min, after which SDS–PAGE gel electrophoresis was used to separate the proteins.

### Blood routine analysis

Peripheral blood from WT mice and *Selenok*-deficient mice were collected into the EDTA vacuum blood collection tubes, and blood routine analysis was carried out with the machine IDEXX Laboratories Inc, ProCyte Dx.

### BioGPS database to analyze SELENOK expression in mice

The BioGPS database (http://biogps.org) was used to analyze the expression profiles of SELENOK in different tissue and cell lines [46].

## Statistical analysis

Statistical analyses were performed using the GraphPad Prism 8 software. Prior to analyzing the statistical significance, we tested the data distribution of the quantitative measurements, and they all conformed to a normal distribution. To compare the data between the two groups, an unpaired two-tailed Student’s t-test was performed. Kaplan–Meier method was used to compare the survival curves. Data from three experiments are presented as mean ± standard deviation (SD). Statistical significance was set at P < 0.05.

## Supporting information

S1 Fig The effects of endoplasmic reticulum-resident selenoproteins on cGAS-STING signaling. **(A-H)** Real-time PCR analysis of *Ifnb* mRNA levels after transfection with the indicated siRNAs for 48h (left) and knockdown efficiency (right) in PMs stimulated with HSV-1. Statistical significance was determined using unpaired two-sided multiple Student’s t-tests. Data represent mean ± standard deviation (SD) or one representative image from three independent experiments. ns: not significant, *: P < 0.05, **: P < 0.01, ***P: < 0.001. siNC, control siRNA; si*Selenok*, si*Dio2*, si*Selenof*, si*Selenom*, si*Selenon*, si*Selenos*, si*Selenot*, si*Selenoi* (S1 Table).
(TIF)

S2 Fig Generation of *Selenok*-deficient mice. **(A)** Relative gene expression levels of *Selenok* in different tissues of mice were analyzed by the BioGPS database (#1423225). **(B)** Relative gene expression levels of *Selenok* in different cell types of mice by the BioGPS database (#1423225). **(C)** Schematic illustration of the target region in *Selenok*^-/-^ mice. The mouse *Selenok* gene (Gene ID:80795) is located on chromosome 14 and contains five exons with an ATG start codon in exon 1 and a TGA stop codon in exon 5. The exons 2–4 were selected as the target sites for deletion. Exons are indicated in blue. **(D)** Schematic illustration of the primers designed to confirm *Selenok* exon deletion. **(E)** PCR analysis of *Selenok* deletion in the genome of WT or *Selenok*^-/-^ extracted from mouse tails. Left panel (Primer-F and Primer-WT-R were used for PCR analysis): WT,437 bp; *Selenok*^-/-^, no product. Right panel (Primer-F and Primer-MT-R were used for PCR analysis): WT, no product; *Selenok*^-/-^:884bp. **(F)** Real-time PCR analysis of *Selenok* mRNA levels in PMs from WT and *Selenok*^-/-^ mice. **(G)**Immunoblot analysis of SELENOK expression in PMs from WT or *Selenok*^-/-^ mice. **(H)** CCK8 analysis of PMs from WT or *Selenok*-deficient mice. **(I)** The picture of WT mice and *Selenok*-deficient mice. **(J)** Blood routine analysis of peripheral blood from WT or *Selenok*-deficient mice. Statistical significance was determined using unpaired two-sided multiple Student’s t-tests in F, H, and J. Data represent mean ± standard deviation (SD) or one representative image from three independent experiments. ns: not significant, ***P: < 0.001.
(TIF)

S3 Fig *Selenok* deficiency has no significant effect on TLR9-triggered innate immune response. **(A and B)** ELISA analysis of IFN-β and IL-6 secretion in PMs following stimulation with CpG ODNs. **(C)** ELISA analysis of IFN-β secretion in BMDMs following stimulation with CpG ODNs. Statistical significance was determined using unpaired two-sided multiple Student’s t-tests in A, B, and C. Data represent mean ± standard deviation (SD) or one representative image from three independent experiments. ns: not significant.
(TIF)

S4 Fig *Selenok* knockdown inhibits STING-triggered innate immune response. **(A)** ELISA analysis of IFN-β secretion in PMs stimulated with DMXAA or cGAMP after transfection with control siRNA (siNC) or si*Selenok* (si*Selenok*-mix). **(B)** Real-time PCR analysis of *Ifnb* mRNA levels in PMs stimulated with DMXAA or cGAMP after transfection with control siRNA (siNC) or *Selenok* siRNA (si*Selenok*-mix). **(C)** Immunoblot analysis of p-IRF3, p-STAT1, and Viperin in PMs stimulated with cGAMP after transfection with control siRNA (siNC) or *Selenok* siRNAs (si*Selenok*-mix). **(D)** Immunoblot analysis of p-IRF3, p-TBK1, p-STAT1, and Viperin in PMs stimulated with DMXAA after transfection with control siRNA (siNC) or *Selenok* siRNA (si*Selenok*-mix). Statistical significance was determined using unpaired two-sided multiple Student’s t-tests in A and B. Data represent mean ± standard deviation (SD) or one representative image from three independent experiments. *: P < 0.05, **P: < 0.01, ***P: < 0.001. siNC, control siRNA; si*Selenok-*mix (S1 Table).
(TIF)

S5 Fig Schematic representation of the role of SELENOK in maintaining STING oligomerization and controlling STING activity. SELENOK facilitated STING-dependent innate immune response by promoting STING oligomerization. Viral infection and IFN-β secretion induced SELENOK expression in the virus-infected cells, and SELENOK then feedback-promoted STING activation. SELENOK deficiency attenuated STING oligomerization, resulting in the inhibition of STING trafficking from the ER to the Golgi and suppression of STING activation.
(TIF)

S1 Table Sequences of siRNA used in this study. (PDF)

S2 Table Sequences of PCR primers used in this study. (PDF)

S1 Data Raw data. Excel spreadsheet containing, in separate sheets, the underlying numerical data and statistical analysis for Figs 1A, 1B, 1C, 1G, 1H, 1I, 1J, 1K, 2B, 2C, 2F, 2G, 3A, 3B, 3C, 3D, 3E, 3F, 3I, 3J, 3K, 3L, 3M, 4A, 4D, 4E, 4F, 4G, 4H, 4K, 4L, 4M, 5A, 5E, 5F, 6G, 6I, 7A, 7B, 7G, 7H, S1, S2A, S2B, S2F, S2H, S2J, S3A, S3B, S3C, S4A, and S4B.
(XLSX)
