# Supplementary figures and images for: Selenoprotein K enhances STING oligomerization to facilitate antiviral response
Source: PLoS Pathog. 2023 Apr 6;19(4):e1011314. doi: 10.1371/journal.ppat.1011314 (PMC10112805; doi:10.1371/journal.ppat.1011314)

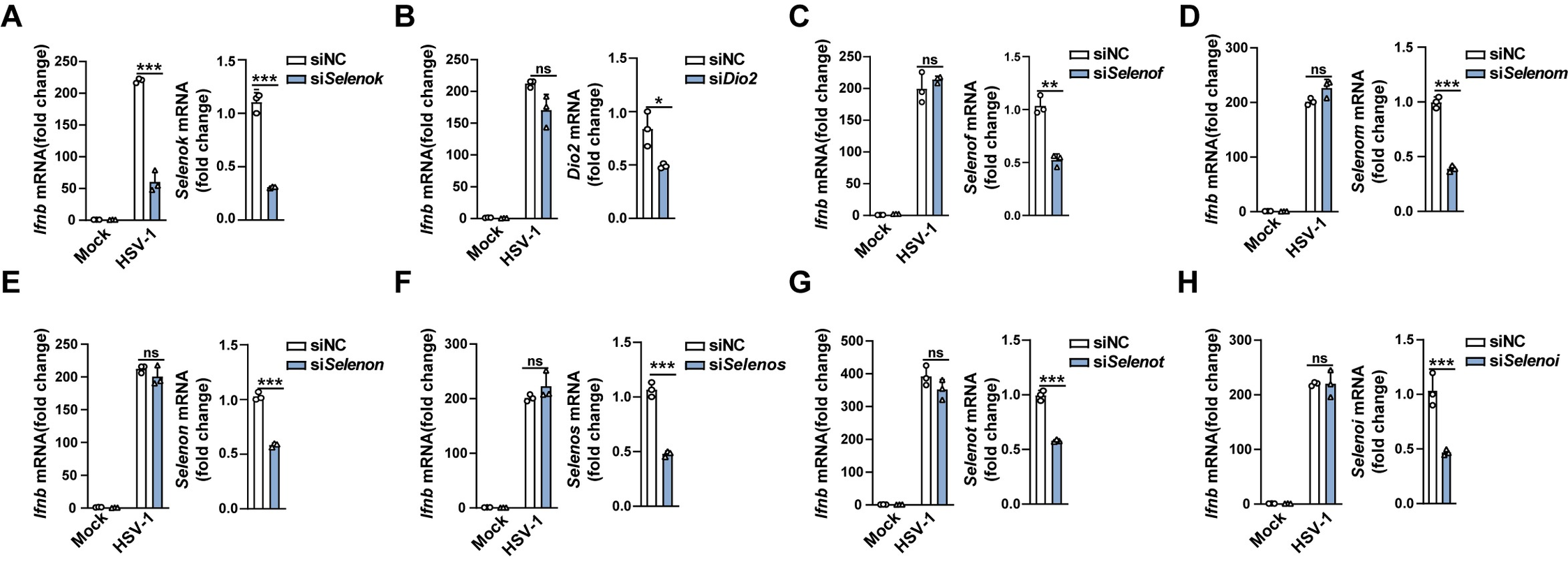

Supplement: S1 Fig — (A-H) Real-time PCR analysis of Ifnb mRNA levels after transfection with the indicated siRNAs for 48h (left) and knockdown efficiency (right) in PMs stimulated with HSV-1. Statistical significance was determined using unpaired two-sided multiple Student’s t-tests. Data represent mean ± standard deviation (SD) or one representative image from three independent experiments. ns: not significant, *: P < 0.05, **: P < 0.01, ***P: < 0.001. siNC, control siRNA; siSelenok, siDio2, siSelenof, siSelenom, siSelenon, siSelenos, siSelenot, siSelenoi (S1 Table). (TIF) [file ppat.1011314.s001.tif]

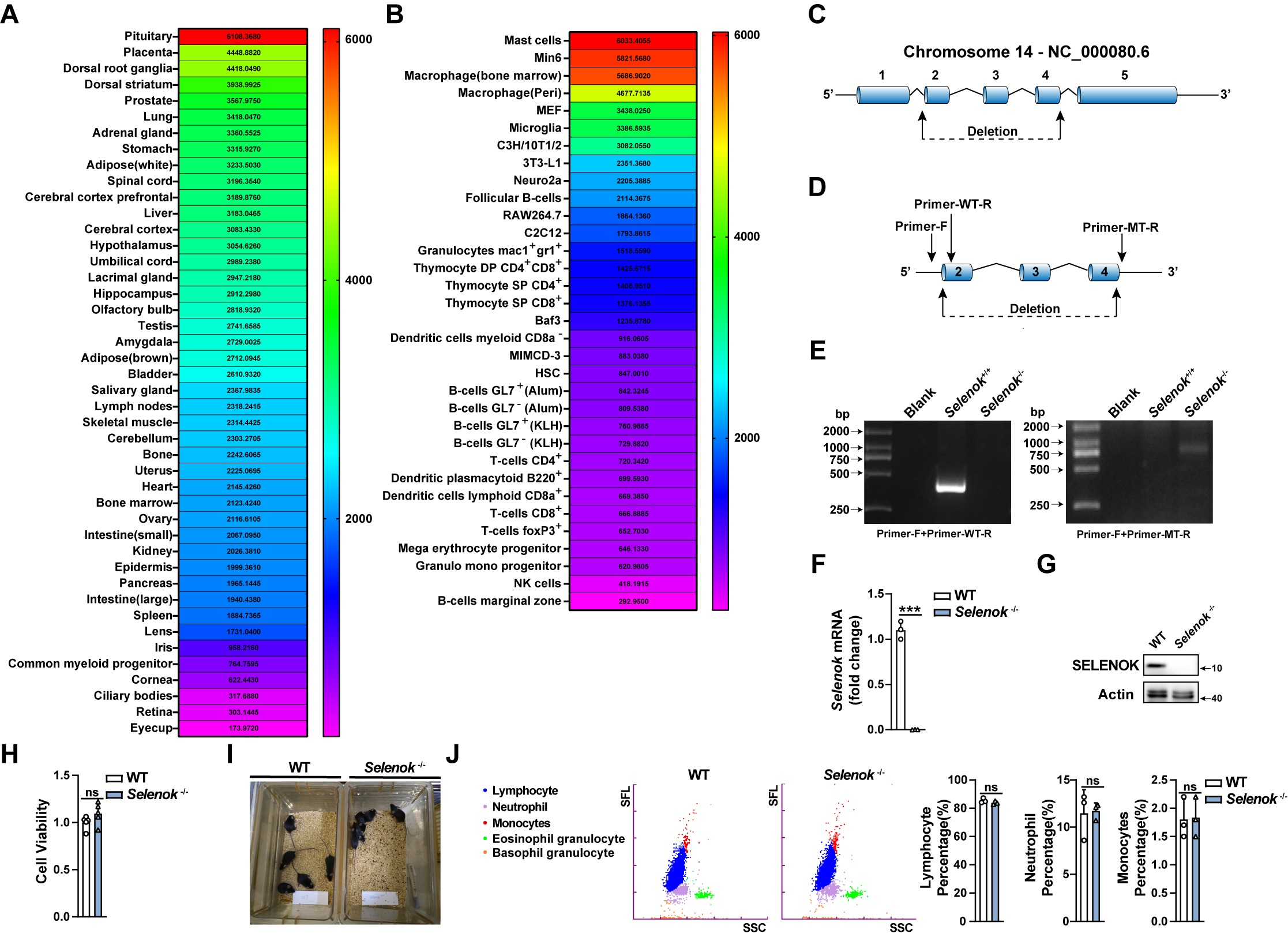

Supplement: S2 Fig — (A) Relative gene expression levels of Selenok in different tissues of mice were analyzed by the BioGPS database (#1423225). (B) Relative gene expression levels of Selenok in different cell types of mice by the BioGPS database (#1423225). (C) Schematic illustration of the target region in Selenok-/- mice. The mouse Selenok gene (Gene ID:80795) is located on chromosome 14 and contains five exons with an ATG start codon in exon 1 and a TGA stop codon in exon 5. The exons 2–4 were selected as the target sites for deletion. Exons are indicated in blue. (D) Schematic illustration of the primers designed to confirm Selenok exon deletion. (E) PCR analysis of Selenok deletion in the genome of WT or Selenok-/- extracted from mouse tails. Left panel (Primer-F and Primer-WT-R were used for PCR analysis): WT,437 bp; Selenok-/-, no product. Right panel (Primer-F and Primer-MT-R were used for PCR analysis): WT, no product; Selenok-/-:884bp. (F) Real-time PCR analysis of Selenok mRNA levels in PMs from WT and Selenok-/- mice. (G)Immunoblot analysis of SELENOK expression in PMs from WT or Selenok-/- mice. (H) CCK8 analysis of PMs from WT or Selenok-deficient mice. (I) The picture of WT mice and Selenok-deficient mice. (J) Blood routine analysis of peripheral blood from WT or Selenok-deficient mice. Statistical significance was determined using unpaired two-sided multiple Student’s t-tests in F, H, and J. Data represent mean ± standard deviation (SD) or one representative image from three independent experiments. ns: not significant, ***P: < 0.001. (TIF) [file ppat.1011314.s002.tif]

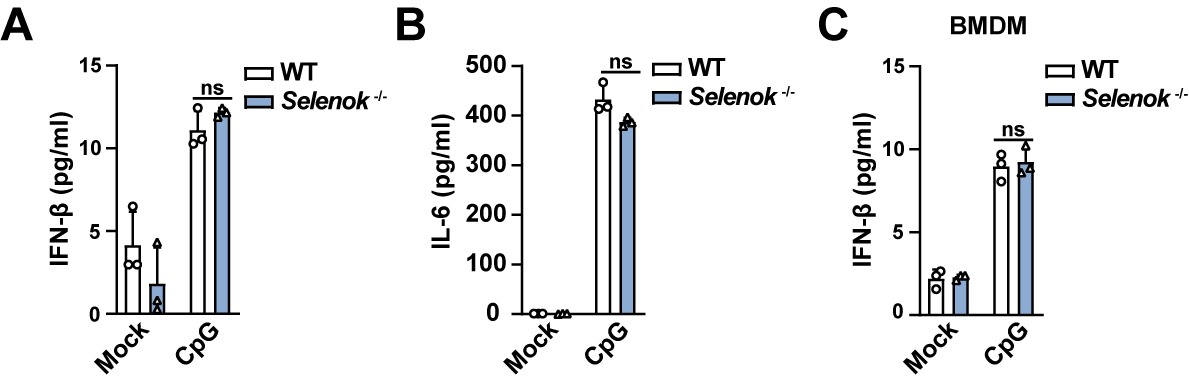

Supplement: S3 Fig — (A and B) ELISA analysis of IFN-β and IL-6 secretion in PMs following stimulation with CpG ODNs. (C) ELISA analysis of IFN-β secretion in BMDMs following stimulation with CpG ODNs. Statistical significance was determined using unpaired two-sided multiple Student’s t-tests in A, B, and C. Data represent mean ± standard deviation (SD) or one representative image from three independent experiments. ns: not significant. (TIF) [file ppat.1011314.s003.tif]

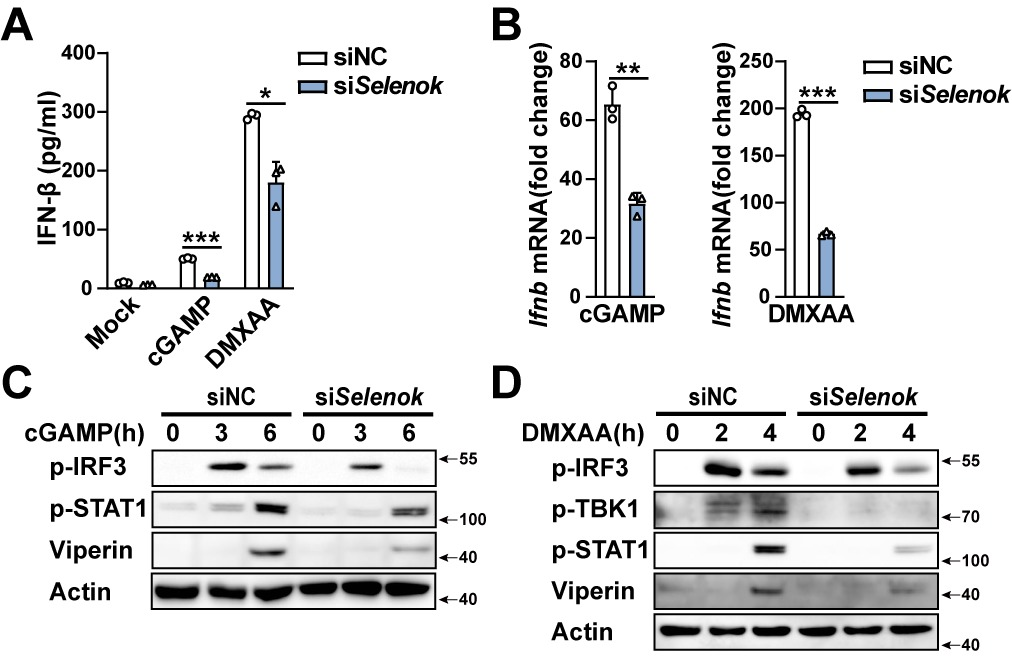

Supplement: S4 Fig — (A) ELISA analysis of IFN-β secretion in PMs stimulated with DMXAA or cGAMP after transfection with control siRNA (siNC) or siSelenok (siSelenok-mix). (B) Real-time PCR analysis of Ifnb mRNA levels in PMs stimulated with DMXAA or cGAMP after transfection with control siRNA (siNC) or Selenok siRNA (siSelenok-mix). (C) Immunoblot analysis of p-IRF3, p-STAT1, and Viperin in PMs stimulated with cGAMP after transfection with control siRNA (siNC) or Selenok siRNAs (siSelenok-mix). (D) Immunoblot analysis of p-IRF3, p-TBK1, p-STAT1, and Viperin in PMs stimulated with DMXAA after transfection with control siRNA (siNC) or Selenok siRNA (siSelenok-mix). Statistical significance was determined using unpaired two-sided multiple Student’s t-tests in A and B. Data represent mean ± standard deviation (SD) or one representative image from three independent experiments. *: P < 0.05, **P: < 0.01, ***P: < 0.001. siNC, control siRNA; siSelenok-mix (S1 Table). (TIF) [file ppat.1011314.s004.tif]

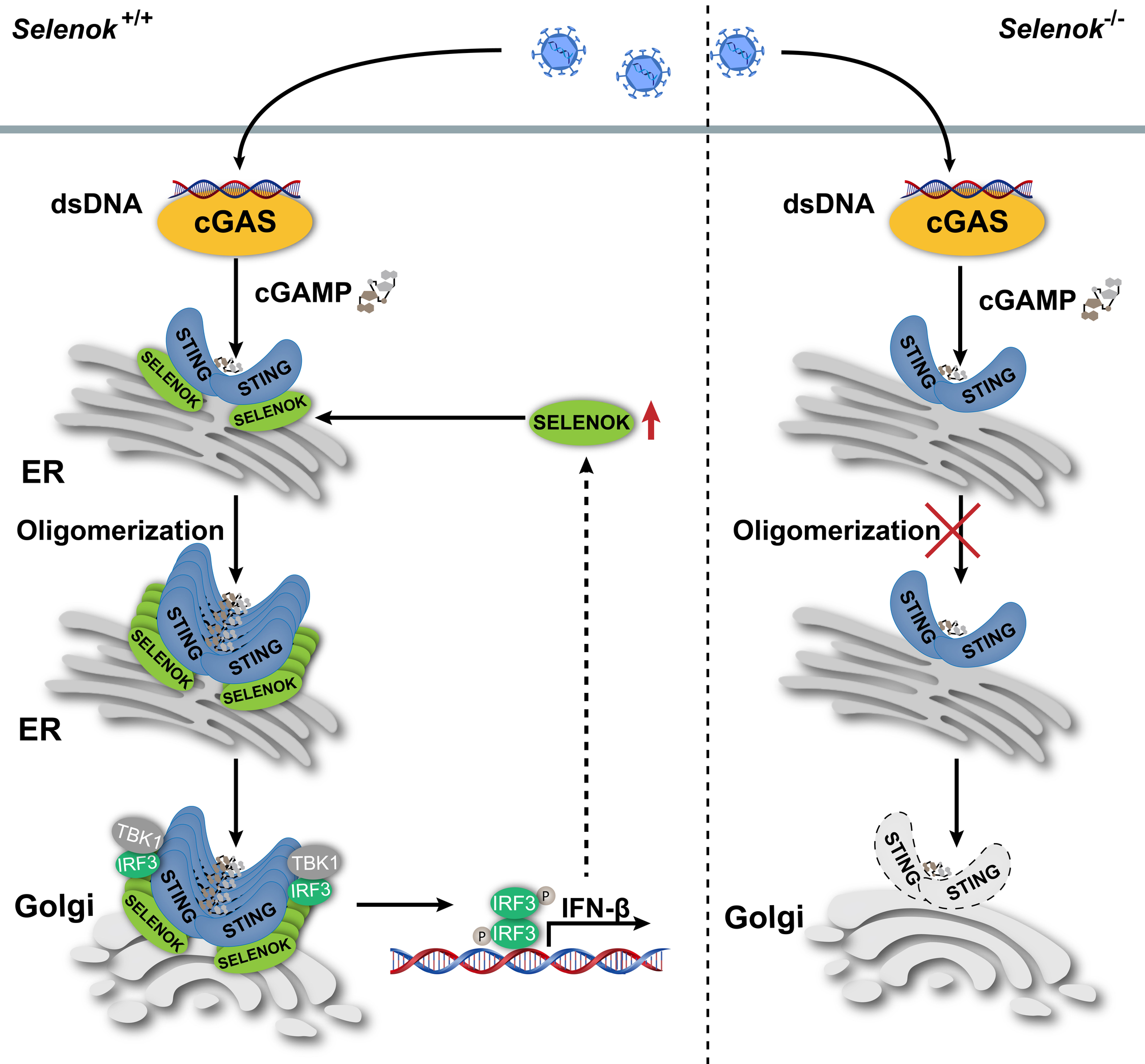

Supplement: S5 Fig — SELENOK facilitated STING-dependent innate immune response by promoting STING oligomerization. Viral infection and IFN-β secretion induced SELENOK expression in the virus-infected cells, and SELENOK then feedback-promoted STING activation. SELENOK deficiency attenuated STING oligomerization, resulting in the inhibition of STING trafficking from the ER to the Golgi and suppression of STING activation. (TIF) [file ppat.1011314.s005.tif]
