# Supplementary material for: Selenoprotein K enhances STING oligomerization to facilitate antiviral response
Source: PLoS Pathog. 2023 Apr 6;19(4):e1011314. doi: 10.1371/journal.ppat.1011314 (PMC10112805; doi:10.1371/journal.ppat.1011314)
Supplement: S1 Table — (PDF) [file ppat.1011314.s006.pdf]

S1 Table. Sequences of siRNA used in this study.

Name	Sequence
“scrambled” control	5’- UUCUCCGAACGUGUCACGUTT -3’
Mouse-*Selenok*-1	5’-UCUCGAAUGGUCAGGUGUUTT-3’
Mouse-*Selenok*-2	5’-CUCUGCUUCAGCAAGAUGUTT-3’
Mouse-*Selenok*-3	5’-GGGUAGGAUCAGUCACCUUTT-3’
Mouse-*Dio2*-1	5’-CCUCCUAGAUGCCUACAAATT-3’
Mouse-*Dio2*-2	5’-CCUGUUGGUAUACAUUGAUTT-3’
Mouse-*Dio2*-3	5’-CUGACCGCAUGGACAAUAATT-3’
Mouse-*Selenof*-1	5’-GGAAGAAGCACAAUUUGAATT-3’
Mouse-*Selenof*-2	5’-CAGAAGUGAUAAACCCAAATT-3’
Mouse-*Selenof*-3	5’-CGGGAACAUUGCUGAAGAATT-3’
Mouse-*Selenom*-1	5’-GACAGUUGAAUCGCCUAAATT-3’
Mouse-*Selenom*-2	5’-UCGUGCUGUUAAGCCGAAATT-3’
Mouse-*Selenon*-1	5’-GCGGCAGGAAUCGGCAUUATT-3’
Mouse-*Selenon*-2	5’-GGUUCUCGCCCGGCCAGUUTT-3’
Mouse-*Selenon*-3	5’-GCCUGCACUUGGAGAAGUATT-3’
Mouse-*Selenos*-1	5’-CCAGCUAUGGCUGGUACAUTT-3’
Mouse-*Selenos*-2	5’-UCUGGAACCUGAUGUUGUUTT-3’
Mouse-*Selenot*-1	5’-GCUGCUCAAGUUCCAGAUUTT-3’

Mouse-*Selenoi*-1	5’-GCUGGCUCCCAAUCUUAUATT-3’
Mouse-*Sec13*-1	5’-CUGUUCCUAUGACCGGAAATT-3’
Mouse-*Sec13*-2	5’-GGUCGAGUGUUUAUUUGGATT-3’
