# Supplementary material for: Selenoprotein K enhances STING oligomerization to facilitate antiviral response
Source: PLoS Pathog. 2023 Apr 6;19(4):e1011314. doi: 10.1371/journal.ppat.1011314 (PMC10112805; doi:10.1371/journal.ppat.1011314)
Supplement: S2 Table — (PDF) [file ppat.1011314.s007.pdf]

S2 Table. Sequences of PCR primers used in this study.

Name	Prime	Sequence
mActb	Forward	5’-TGTTACCAACTGGGACGACA-3’
	Reverse	5’-CTGGGTCATCTTTTCACGGT- 3’
mIfnb	Forward	5’-ATGAGTGGTGGTTGCAGGC- 3’
	Reverse	5’-TGACCTTTCAAATGCAGTAGATTCA3’
mIl-6	Forward	5’-ACAACCACGGCCTTCCCTAC-3’
	Reverse	5’-CATTTCCACGATTTCCCAGA-3’
mCxcl10	Forward	5’-ATCATCCCTGCGAGCCTATCCT-3’
	Reverse	5’-GACCTTTTTTGGCTAAACGCTTTC-3’
mIsg15	Forward	5’-AGAAGCAGATTGCCCAGAAG-3’
	Reverse	5’-TGCGTCAGAAAGACCTCATAGA-3’
mIsg54	Forward	5’-CCTAAACAGTTACTCCACCTTCG-3’
	Reverse	5’-TTGCTGACCTCCTCCATTCT-3’
mRantes	Forward	5’-CACCACTCCCTGCTGCTTTG-3’
	Reverse	5’-ACACTTGGCGGTTCCTTCG-3’
HSV-1 *UL30*	Forward	5’-CATCACCGACCCGGAGAGGGAC-3’
	Reverse	5’-GGGCCAGGCGCTTGTTGGTGTA-3’
mSelenok	Forward	5’-GTTTACATCTCGAATGGTCAG G-3’
	Reverse	5’-CCACCAGCCATTGGAGGAGGGC-3’
mDio2	Forward	5’-CACCGGCTCTATTCTTCATACC-3’

	Reverse	5’-GTTCAGACTCACCTTGGAACA-3’
mSelenof	Forward	5’-CTCACCAGTGAAACGCTTTG-3’
	Reverse	5’-TCAAAGAGCACACAGCAAGG-3’
mSelenom	Forward	5’-TCGCCTAAAGGAGGTGAAGG-3’
	Reverse	5’-GGTCATTTGGCTGAGTGGGA-3’
mSelenon	Forward	5’-TGTTGACCTGATGACCCAAG -3’
	Reverse	5’-CTAGACAGTGCTGGCAATAAGA-3’
mSelenos	Forward	5’-ATCATCTGGCGGCTGAAACT-3’
	Reverse	5’-GAGAAAAGCCCCACCTCTCC-3’
mSelenot	Forward	5’-TTAAATGATGTGCCAGTGTGGT-3’
	Reverse	5’-ATGTCAGTCACGCTGCTCTT-3’
mSelenoi	Forward	5’-TGCTTCAGCCCCTGGTCATA-3’
	Reverse	5’-AACACACGACCAACTGTCCA-3’
Primer for WT	Forward	5’-GGGCACTACAGGAAGCATCATC-3’
	Reverse	5’-TGCTGTCCAACACCTGACCTAC-3’
Primer for MT	Forward	5’-GGGCACTACAGGAAGCATCATC-3’
	Reverse	5’-GAAGCCAAGGAGGTCCAAGGAG-3’
